# Economic Evaluation of a Novel MicroRNA-Based Assay to Determine Risk of Late Genitourinary Radiation Toxicity in Patients With Prostate Cancer

**DOI:** 10.36469/001c.146844

**Published:** 2025-12-16

**Authors:** Jacie T. Cooper, John E. Schneider

**Affiliations:** 1 Avalon Health Economics

**Keywords:** toxicity, radiation, microRNA, prostate cancer

## Abstract

**Background:**

Prostate cancer is one of the most common malignancies in men, and radiation therapy, most often stereotactic body radiation therapy or conventionally fractionated radiotherapy, is a standard curative treatment for advanced cases. Although genitourinary toxicity is a known side effect, with acute symptoms typically resolving and late toxicity causing lasting harm, individual risk varies substantially. However, no validated tool exists to predict patient-specific toxicity across radiation modalities, leaving treatment decisions to be made without personalized risk insights.

**Objective:**

The goal of this analysis was to estimate changes in cost and quality of life associated with reductions in late genitourinary toxicity attributable to personalized radiation therapy in individuals with prostate cancer. Implementation of PROSTOX *ultra*, a novel microRNA-based assay for toxicity risk assessment with stereotactic body radiation therapy, is expected to enable the personalization of radiation therapy, helping patients avoid toxic effects from specific types of radiation therapy.

**Methods:**

This study utilizes a hybrid decision-tree and Markov model approach to estimate the 5-year cost-impact and lifetime cost-effectiveness in prostate cancer patients of PROSTOX *ultra* vs current treatment standards that do not include toxicity risk assessment. High or low risk assessment influences whether patients receive stereotactic body radiation therapy, conventionally fractionated radiotherapy, or prostatectomy, impacting toxicity-related treatment costs and quality-of-life decrements.

**Results:**

Over 5 years, PROSTOX *ultra* patients totaled 47 683incostsvs67 298 with standard-care risk assessment. Cumulative savings over 5 years were $19 615 per tested patient. Over a lifetime, PROSTOX *ultra* was expected to save about $24 777 per tested patient while adding 0.24 quality-adjusted life-years compared with patients tested using standard assessment.

**Conclusions:**

Model results indicate that toxicity risk assessment with PROSTOX *ultra* would be cost saving over 5 years compared with standard risk assessment. Cost-effectiveness results show cost savings and quality-adjusted life-year gains over a lifetime, indicating that PROSTOX *ultra* would be a dominant strategy compared with treatment without risk assessment.

## INTRODUCTION

Prostate cancer is one of the most common cancers in men worldwide and is the cause of 3.8% of all male cancer deaths.^1^ Screening for prostate cancer usually starts at age 50 years, and the average age at diagnosis is 67 years.^2^ For patients with low-risk cancer, active surveillance is a reasonable strategy to avoid treatment and the aligning side effects.^3^ However, for patients with progressed cancer warranting curative treatment intervention, radiation therapy and surgical prostatectomy are the most common approaches.^4^ Both interventions are associated with risk of urinary tract complications, which can have significant negative impacts on quality of life (QoL).^5,6^ For radiation treatment specifically, patients may experience either acute (within 90 days of treatment start) or late (>90 days from treatment start) genitourinary (GU) toxicity. While acute toxicity is usually temporary, late toxicity is associated with long-term damage, therefore causing long-lasting symptoms.^7^

The most utilized types of external beam radiation therapy for prostate cancer are stereotactic body radiation therapy (SBRT) and conventionally fractionated radiotherapy (CFRT). SBRT delivers high doses of radiation treatment in 5 or fewer fractions, resulting in a shorter, less expensive, patient-preferred treatment approach. By contrast, CFRT delivers between 35 and 44 low-dose fractions over a longer period.^8^ Both approaches have similar efficacy for improving prostate cancer cure and overall survival for prostate cancer patients.

While there is a slightly higher risk for toxicity with SBRT compared with CFRT, risk varies uniquely for each patient, and patients may be at high risk of toxic effects for one type of radiation but low risk for the other.^9^ There currently is no tool utilized for toxicity prediction with radiation therapies. Therefore, patient treatment pathways are assigned without knowledge of their personal toxicity risk profile. To combat this, MiraDx has developed PROSTOX™ *ultra*, an assay that analyzes a patient’s germline DNA to determine if the individual is at risk of late GU radiation toxicity based on treatment approach (ie, SBRT vs CFRT). The test provides a high or low risk score that can be used to assign the safest course of treatment to avoid toxicity. A recent validation study has confirmed the clinical utility of PROSTOX *ultra* to personalize radiation therapy.^10^

This analysis focuses on the value of personalized cancer treatment informed by PROSTOX *ultra*, which specifically tests patients for risk of late GU radiation toxicity from SBRT. PROSTOX *ultra* has a reported positive predictive value of 0.64 and a negative predictive value of 0.96 (sensitivity, 0.79; specificity, 0.92).^9^ Implementation of PROSTOX *ultra* is expected to enable the personalization of radiation, helping patients avoid toxicity from specific types of radiation therapy. In doing so, patients can experience fewer toxicity-related treatment costs and avoid QoL decrements associated with GU toxicity.

This study presents the methods and results of a lifetime cost-effectiveness analysis and a 5-year cost-impact analysis employed to capture the expected value to US payers of PROSTOX *ultra* utilization in prostate cancer patients. Results may help decision-makers evaluate the costs and benefits of accurately assessing the risk of late GU radiation toxicity from SBRT to guide treatment decisions. The goal of this analysis was to estimate the US payer-perspective cost savings and QoL improvements associated with reductions in late GU toxicity enabled by personalized radiation therapy approaches with PROSTOX *ultra*.

## METHODS

## Population

The analysis was conducted in Microsoft Excel. The modeled population consisted of patients with diagnosed prostate cancer who had been recommended for active treatment. It was assumed that no patients in this cohort were eligible for active surveillance or watchful waiting and that all patients would receive either SBRT, CFRT, or a prostatectomy.

### Comparators and Outcomes

The analysis compared the 5-year cost impact and lifetime cost-effectiveness of PROSTOX *ultra*–guided treatment compared with standard-of-care (SOC) treatment recommendations. The 5-year incremental costs provided insight into the expected short-term economic impacts that PROSTOX *ultra* may have. Meanwhile, the lifetime analysis produced incremental costs, incremental quality-adjusted life-years (QALYs), and an incremental cost-effectiveness ratio (ICER) to compare the long-term cost and clinical outcomes between PROSTOX *ultra* and SOC.

A QALY captures patient QoL and life expectancy into a single measure, representing the total value of health outcomes. A QALY is calculated by multiplying total expected years of life by the average corresponding QoL, measured as a utility value on a scale from 0 (death) to 1 (perfect health). An ICER estimates the economic value of an intervention compared with standard care, using the QALY as the measure of clinical effectiveness. An ICER is calculated as the difference in costs divided by the difference in QALYs between two interventions, generating a cost per QALY. This outcome represents the amount that one would expect to pay with the new intervention to gain 1 year of perfect health.

### Model Framework

The model framework consisted of two interconnected structures. A decision tree was used to capture the first stage of the clinical story, depicting the patient treatment decision pathway. This fed directly into a lifetime Markov simulation, which modeled the clinical and economic toxicity-driven outcomes of each treatment pathway. Discounting was not applied in the model as it was assumed that the rate of inflation would equal the discount rate over the relevant period.

**Figure 1.**
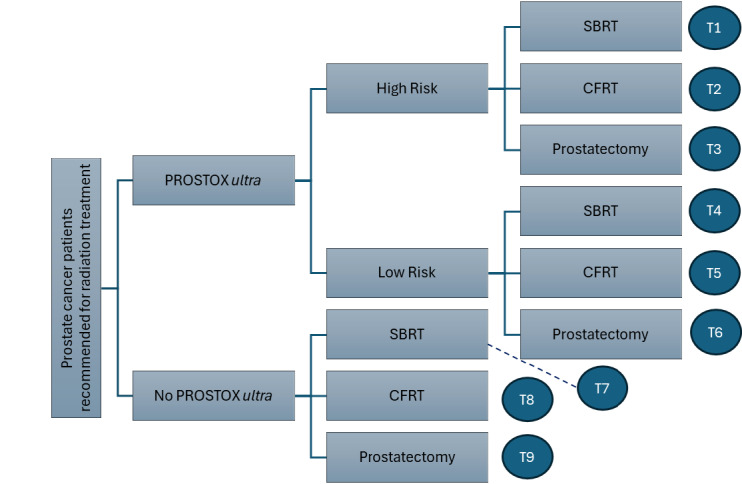
Decision Tree Structure Abbreviations: CFRT, conventionally fractionated radiotherapy; SBRT, stereotactic body radiation therapy; T1, PROSTOX high-risk SBRT; T2, PROSTOX high-risk CFRT; T3, PROSTOX high-risk prostatectomy; T4, PROSTOX low-risk SBRT; T5, PROSTOX low-risk CFRT; T6, PROSTOX low-risk prostatectomy, T7, SOC SBRT; T8, SOC CFRT; T9, SOC prostatectomy.

**Decision tree: Figure 1** displays the decision pathway for patients with treatment guided by PROSTOX *ultra* results compared with patients not utilizing PROSTOX *ultra* (SOC). With SOC, patients either proceeded to receive SBRT, CFRT, or prostatectomy as estimated by general population utilization estimates from the literature. Patients utilizing PROSTOX *ultra*–guided treatment were stratified as high risk or low risk for late GU toxicity from SBRT. This risk assessment impacted the resulting treatment strategy, with fewer high-risk patients receiving SBRT and more patients receiving SBRT when considered low risk.

Each arm of the decision tree is shown alongside a labeled end node, indicating the treatment “track” that patients on that pathway experience. Each of the 9 tracks was associated with unique costs, QoL values, and transition probabilities within the ensuing Markov model.

**Markov model:** Each track’s population fed into a unique Markov model that followed patient toxicity-related outcomes through their lifetime. Each of the Markov models shared an identical model structure but were populated with individual input parameters depending on the population track, which varied the time spent in each disease state and resulting costs and clinical outcomes.

**Figure 2.**
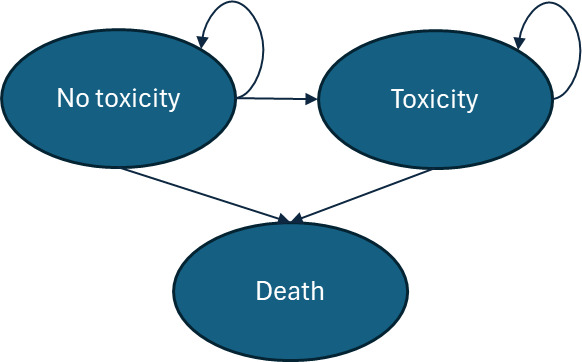
Markov Simulation Structure

Each Markov simulation consisted of three distinct disease states: no-toxicity, toxicity, and death (**Figure 2**). It was assumed that the entire patient population, regardless of track, started the Markov model at age 67 years (the average age of prostate cancer diagnosis in the United States).^11^ Each cycle length was 1 year, and the model continued through the cohort’s lifetime, ending after all patients have moved into the death state. In each track simulation, 10 000 patients were entered into the model. The simulation was assumed to begin at the threshold of the “late toxicity” definition, 90 days after radiotherapy (or surgery) completion. All patients therefore entered the model (*T0*) in the “no-toxicity” state. In cycle 1, patients can move into the “toxicity” disease state, which represents the probability of late toxicity development in the first year after toxicity would meet the “late” toxicity definition. Once a patient moves into the late-toxicity disease state, it was assumed that they will experience the aligning management costs and QoL decrements through the remainder of their life. The death state was assumed to be absorbing (meaning that patients cannot exit this disease state once entering).

It is important to recognize that prostatectomy does not result in what one would consider “toxicity,” as this term generally encompasses effects specific to radiation therapy. However, this model captured the GU side effects of prostatectomy under the “toxicity” definition, as there was significant overlap between the symptoms of radiation-driven toxicity and surgery-caused GU complications. For instance, both treatments can cause symptoms such as urinary frequency, urinary urgency, and incontinence.^12^ For this reason, patients on a surgical track still entered a Markov model with toxicity and no-toxicity disease states.

**Clinical inputs**: All clinical parameters were extracted from targeted literature searches. Decision tree and Markov model–specific inputs are displayed alongside references in **Table 1**. Where assumptions or calculations were utilized, the references column of the table provides a footnote describing the input’s justification. While the impact that PROSTOX *ultra* would have on clinical practice is still being defined, the current model assumed that 100% of patients receiving radiation therapy with PROSTOX *ultra* results would adhere to the radiation modality recommended for their risk outcome. Therefore, no patients receiving a high-risk PROSTOX *ultra* result would receive SBRT, while no patients receiving a low-risk PROSTOX *ultra* result would receive CFRT. The 100% adherence did not apply to patients who would have undergone prostatectomy with SOC. Instead, among the patients who received PROSTOX *ultra* and were low risk, we assumed that, over time, the utilization of prostatectomy would be reduced from about 49% with SOC to about 10%. This represented an 80% decrease in utilization compared with SOC, as most patients were expected to utilize SBRT in place of surgery if not deemed at high risk for radiation toxicity.^13^

**Table 1.** Clinical and Economic Inputs

**Field**	**Value**	**Reference/Assumption**
Decision-tree parameters		
PROSTOX *ultra* patients predicted high risk for GU toxicity	18.72%	Kishan et al^9^
SBRT utilization	0.00%	Assumption^a^
CFRT utilization	50.65%	Calculation^b^
Prostatectomy utilization	49.35%	Wang et al^4^
PROSTOX *ultra* patients predicted not high risk for GU toxicity	81.28%	Kishan et al^9^
SBRT utilization	90.13%	Assumption^c,d^
CFRT utilization	0.00%	Assumption^c^
Prostatectomy utilization	9.87%	Calculation^d^
Standard of care		
SBRT utilization	38.30%	Gao et al^14^
CFRT utilization	12.35%	Gao et al^14^
Prostatectomy utilization	49.35%	Wang et al^4^
Markov model parameters		
Population without GU toxicity at T0	100.00%	Assumption^e^
Probabilities of transitioning from no-toxicity to toxicity cycle 1		
High-risk PROSTOX *ultra* patients using SBRT (track 1)	0.2778	Kishan et al^9^^f^
All patients using CFRT (tracks 2, 5, and 8)	0.0396	van As et al^15^^g^
All patients using prostatectomy (tracks 3, 6, and 9)	0.1189	Donovan et al^16^^h^
Not high risk PROSTOX *ultra* patients using SBRT (track 4)	0.0124	Kishan et al^9^^f^
SOC patients using SBRT (track 7)	0.0499	Kishan et al^9^^f^
Annual toxicity development taper for cycles 5+ (SBRT and CFRT)	0.7013	Kishan et al^17^^i^
Utility without GU toxicity	0.90	Khairnar et al^18^
Utility with GU toxicity	0.70	Schumacher et al^19^^j^
Discount rate for costs	3.00%	Neumann et al^20^
Discount rate for utilities	3.00%	Neumann et al^20^
Average total cost of SBRT	$20 670.28	Hodges et al^21^
Average total cost of CFRT	$42 642.68	Hodges et al^21^
Average total cost of prostatectomy	$97 782.37	HCUPnet^22^^k^
Cost of PROSTOX *ultra* test	$4000.00	List price
Cost of care without GU toxicity (annual)	$2914.90	National Cancer Institute^23^
Cost of care with GU toxicity (annual)	$5774.58	Schumacher et al^19^^l^

Abbreviations: CFRT, conventionally fractionated radiation therapy; GU, genitourinary; SBRT, stereotactic body radiation therapy; SOC, standard of care.

^a^Assume no patients identified as high risk receive SBRT.

^b^Assume all nonsurgical patients utilize CFRT.

^c^Assume no patients utilize CFRT with a low-risk PROSTOX *ultra* result.

^d^Assume 80% of surgical SOC patients (80% of 49.35% = 39.48%) utilize SBRT instead with low-risk PROSTOX *ultra* result; these patients are shifted from the prostatectomy arm into the SBRT arm for low-risk PROSTOX *ultra* patients.

^e^Assume all patients start in the no-toxicity state at t0.

^f^Rate of 3.5-year late toxicity development converted into 1-year probability.

^g^Rate of 5-year late toxicity development converted into 1-year probability.

^h^Utilizing 10- and 5-year cumulative toxicity incidence.

^i^Average of 12- to 72-month EPIC urinary summary scores.

^j^Average grade 2 and grade 3+ toxicity utilities weighted by population size as reported by Mak et al.^24^

^k^Average of 2022 hospital charges for MS-DRGs 665, 666, and 667 weighted by discharge count in 2022.

^l^Average of grades 2 and 3+ annual excess toxicity costs weighted by population size as reported by Mak et al^24^ summed with the average annual cost of care without toxicity from the National Cancer Institute.^23^

All toxicity rates shown in **Table 1** represented the probability of GU toxicity development in the first cycle of the model. For all SBRT and CFRT tracks, this transition probability was maintained until cycle 5, where a toxicity development taper took effect. For all prostatectomy tracks (tracks 3, 6, and 9), patients can only move into the toxicity state in the first cycle of the model. No toxicity development was modeled for this population thereafter, although the model assumed patients remained in the toxicity state until death. Annual toxicity probabilities for each track and calculations are shown in **Supplementary Table S1**.

**Economic inputs**: All cost parameters are displayed in **Table 1** alongside references from targeted literature searches. Economic values were inflated to 2024 US dollars using the Bureau of Labor Statistics Consumer Price Index for medical care as needed.^25^

**Uncertainty analyses**: A one-way deterministic sensitivity analysis (DSA) was conducted to assess the impact of independent variations to input parameters on the overall 5-year cost-impact results. This analysis varied each model parameter by 20% in either direction, estimating results at the 20% high value and the 20% low value. For the lifetime cost-effectiveness model, a probabilistic sensitivity analysis (PSA) was performed to account for uncertainty in model inputs (**Supplementary Table S2**). The PSA consisted of 10 000 model simulations in which each variable was randomly varied along its appropriate distribution curve. Generally, cost inputs were varied along a gamma distribution while probabilities and utilities were varied along a beta distribution. Where error data were not available, a 10% standard error from the mean value was assumed.

## RESULTS

The decision tree model resulted in 9.5% of the PROSTOX *ultra* population in track 2 (high-risk CFRT), 9.2% in track 3 (high-risk prostatectomy), 73.3% in track 4 (low-risk SBRT), and 8.0% in track 6 (low-risk prostatectomy). No patients followed tracks 1 or 5 (high-risk SBRT, low-risk CFRT). In the SOC arm, 38.3% of patients were in track 7 (SBRT), 12.4% in track 8 (CFRT), and 49.4% in track 9 (prostatectomy). These distributions equate to 73.3% of the total PROSTOX *ultra* population receiving SBRT, 9.5% receiving CFRT, and 17.3% undergoing prostatectomy, compared with 38.3% of SOC patients receiving SBRT, 12.4% receiving CFRT, and 49.4% undergoing prostatectomy.

The annual per-patient costs and QALY outcomes for each track are shown in **Table 2** alongside the track weight, which represents the percentage of each arm’s population entering each track of the model. Treatment costs were assumed to be accumulated at the start of the Markov model and were therefore presented separately from the annual clinical costs. The presented annual costs were specific to each population track; for instance, 38.30% of SOC patients experienced the per-patient annual costs (shown in **Table 2** for the first 5 years of the model) and aligning lifetime costs and QALYs listed for track 7 (SOC SBRT).

**Table 2.** Per-Patient Outcomes by Treatment Track

**Track**	**Track Weight, %**	**Treatment Costs (T0), $**	**Annual Clinical Costs, $**					**Lifetime Outcomes**	
**Year 1**	**Year 2**	**Year 3**	**Year 4**	**Year 5**	**Costs**	**QALYs**			
PROSTOX *ultra* (high risk)									
T1 (SBRT)	0.00	20 670	0	0	0	0	0	24 170	0.00
T2 (CFRT)	9.48	42 643	276	274	271	266	260	93 306	11.28
T3 (prostatectomy)	9.24	97 782	270	287	274	260	247	144 030	11.59
PROSTOX *ultra* (low risk)									
T4 (SBRT)	73.26	20 670	2135	2061	1987	1911	1836	65 700	11.68
T5 (CFRT)	0.00	42 643	0	0	0	0	0	46 143	0.00
T6 (prostatectomy)	8.02	97 782	234	250	238	226	215	144 030	11.59
SOC									
T7 (SBRT)	38.30	20 670	1117	1118	1112	1101	1085	69 806	11.15
T8 (CFRT)	12.35	42 643	360	357	352	346	339	89 806	11.28
T9 (prostatectomy)	49.35	97 782	1439	1534	1461	1390	1320	140 530	11.59

Abbreviations: CFRT, conventionally fractionated radiation therapy; GU, genitourinary; QALY, quality-adjusted life-year; SBRT, stereotactic body radiation therapy; SOC, standard of care.

### Five-Year Cost Impact

Cost-impact results were estimated by weighting each track’s corresponding costs by the percentage of the population in each arm. For instance, **Table 2** shows year 5 costs for SOC patients to be $1085 for patients in track 7 (SBRT; 38.30% of SOC patients), $339 for patients in track 8 (CFRT; 12.35% of SOC patients), and $1320 for patients in track 9 (prostatectomy; 49.35% of SOC patients). The year 5 costs for SOC patients were therefore calculated as the sum-product of the costs and the percentage of the SOC population in each arm ($1109). Using the same approach for each year (including T0) and summing across all annual totals yielded the cumulative 5-year costs.

**Figure 3.**
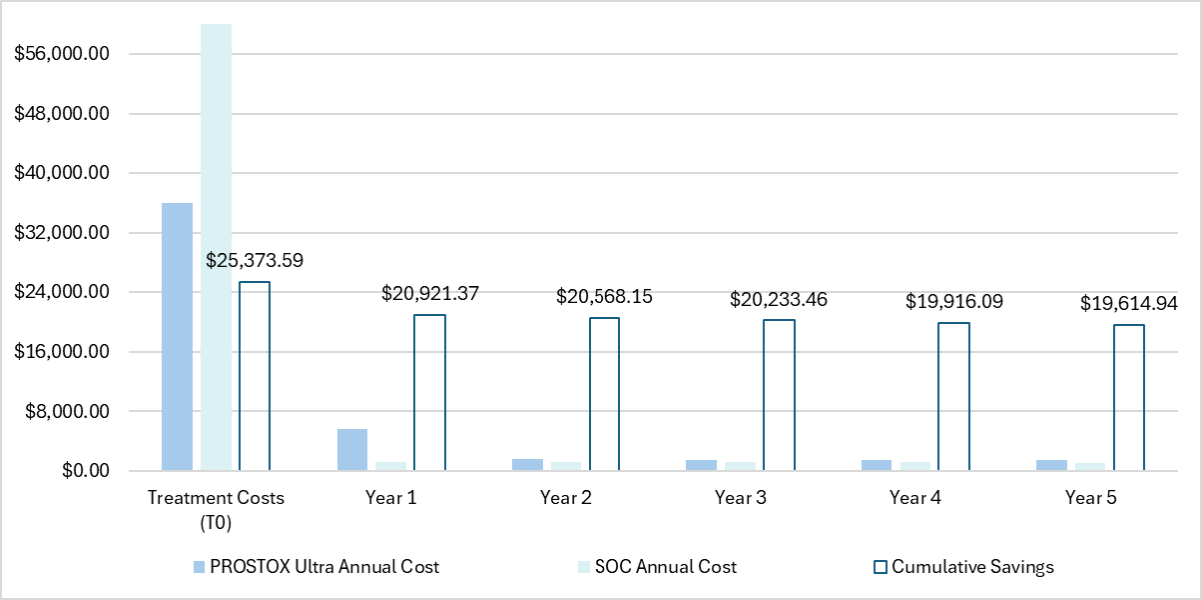
Annual Costs and Cumulative Savings Over 5 Years Abbreviation: SOC, standard of care.

The resulting annual costs and cumulative savings through a 5-year period are depicted for each arm in **Figure 3**. Over 5 years, patients using PROSTOX *ultra* totaled $47 683 in costs, compared with costs of $67 298 with SOC. Cumulative savings over 5 years were therefore estimated to be $19 615 per tested patient. When running the model at various costs for PROSTOX *ultra*, positive savings were still seen even if priced over $10 000.

According to the DSA, the variable with the greatest impact on 5-year cost savings was the percentage of patients in SOC receiving prostatectomy, followed closely in scale by the cost of the prostatectomy (**Supplementary Figure S1**). This is logical given the high cost of surgery. Similarly influential parameters were the percentage of SOC surgical patients who would instead use SBRT with a low-risk PROSTOX *ultra* result and the adherence to the PROSTOX *ultra* result for SOC radiation patients. As these are the model assumptions most likely to vary unpredictably in the real-world setting, it is significant that savings remained prominent with changes to these values. Most importantly, this analysis confirms that the savings generated in the cost-impact model remain robust to variations in all modeled input parameters.

### Lifetime Cost-Effectiveness Analysis

In the cost-effectiveness analysis, there was a noticeable differential in total cohort QALYs (11.63 vs 11.39), which was likely driven by the increased utility values for patients in PROSTOX *ultra* track 4 (low-risk patients using SBRT, who have a reduced risk of toxicity compared with SOC patients). There was no life-year difference between the two arms, as the model did not model an effect on mortality. PROSTOX *ultra* had lower lifetime costs compared with SOC ($82 337 vs $107 114), thus indicating a dominant strategy (incremental cost-effectiveness ratio) with QALY gains of 0.24 and cost savings of $24 777.

**Figure 4.**
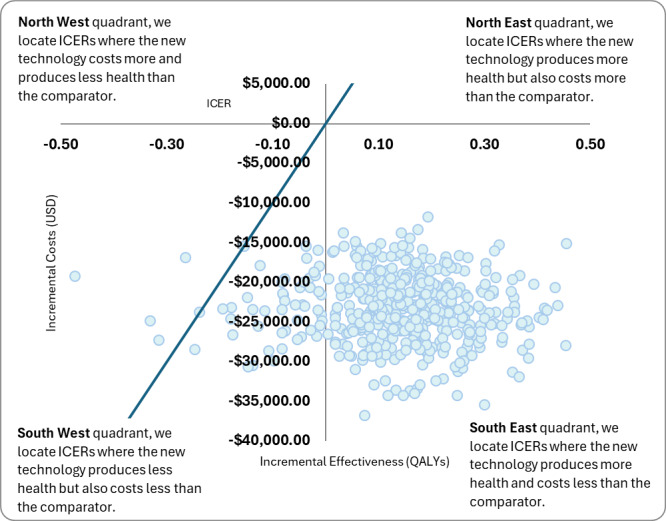
Probabilistic Sensitivity Analysis Scatterplot Abbreviations: ICER, incremental cost-efficiency ratio; QALY, quality-adjusted life-year.

A scatterplot of the outcomes of each simulation run of the PSA is shown in **Figure 4**. A trendline representing the standard willingness-to-pay threshold in the United States ($100 000 per QALY) was plotted for reference. Nearly all the simulation datapoints fall below this threshold line and are thus considered cost-effective. Most of the simulation ICERs fall into the southeast quadrant of the cost-effectiveness plane, which depicts a dominant strategy. Plotting of cost-effectiveness acceptability curve (**Supplementary Figure S2**) for PROSTOX *ultra* and SOC showed that PROSTOX *ultra* would be cost-effective at any willingness-to-pay threshold through $150 000, which is logical as it is a dominant strategy compared with SOC.

## DISCUSSION

This analysis emphasized the economic benefits of adapting PROSTOX *ultra* as a prediction tool for risk of late GU toxicity with SBRT in patients with prostate cancer. Utilizing PROSTOX *ultra* in place of the current SOC decision-making process, which does not include a risk-ranking tool, was expected to save $19 615 per tested patient over a 5-year period. PROSTOX *ultra* was also expected to reduce overall lifetime costs and increase QoL compared with SOC.

The value of PROSTOX *ultra* stems first from allocating proper radiation technology to each patient according to their toxicity risk. As supported by a study conducted to assess whether PROSTOX *ultra* would influence treatment choice, patients at high risk of toxicity from SBRT would utilize CFRT in its place, resulting in a decreased comparative risk of toxicity, while patients with a low risk of toxicity with SBRT would logically utilize SBRT.^26^ Further, patients with a low risk score may be encouraged to utilize SBRT radiation therapy instead of proceeding with a prostatectomy, given the low risk of toxicity-related side effects.^13^ The value identified in this analysis is expected to persist when considering utilization of moderately hypofractionated radiation therapy vs CFRT as well, as toxicity outcomes are similar between these two therapies.^27^

This evidence is especially relevant for clinicians making treatment decisions for patients with prostate cancer, as both improvements in patient QoL and reductions in payer costs are presented. Recent studies have shown the utilization of short-form radiation therapy rising over time, implying that the results of this SBRT study may become increasingly relevant in future years.^28^

As PROSTOX *ultra* is, to our knowledge, the first risk-ranking predictive tool for radiation therapy in the prostate cancer space, direct comparison to existing economic analyses was not possible. However, evidence has been recently published assessing the cost-effectiveness of gene expression profiling tests for breast cancer relapse risk to guide the utilization of endocrine therapy and adjuvant chemotherapy. A 2025 analysis of the *Prosigna* assay in Norway identified a non-cost-effective ICER of €255 622 compared with standard immunohistochemical markers but showed cost-effectiveness in a population restricted to patients assessed as uncertain chemotherapy candidates (ICER, €8884).^29^

This study has three important limitations. First, the analysis was a cohort-driven model that represents the average patient with prostate cancer and did not reflect specific subgroups or unique cases. However, because PROSTOX *ultra* is based not on tumor analysis, but on inherited genes, subgroups and unique cases would not be expected to alter the conclusions. Second, most of the cost and utilization parameters were literature based and were therefore subject to heterogeneity in the respective populations. Finally, the model was driven by assumptions on PROSTOX *ultra* treatment adherence and influence. While the expected impact of PROSTOX *ultra* was supported by evidence of its prediction accuracy, the actual clinical impacts were unknown as the adherence to PROSTOX *ultra*’s outcomes is evolving. A current unpublished clinical study estimates 80% compliance to alternative treatment instead of SBRT with a high-risk PROSTOX *ultra* result, and trust in the tool’s risk ranking is expected to rise over time, increasing adherence to test results as clinician confidence continues to grow.

## CONCLUSIONS

Utilization of PROSTOX *ultra* to determine risk of late GU radiation toxicity from SBRT was expected to generate short-term cost savings due to reductions in toxicity development and fewer costly prostatectomies. The implementation of PROSTOX *ultra* was estimated to save $19 615 per tested patient over a 5-year period. The analysis also supported the cost-effectiveness of PROSTOX *ultra* compared with SOC, predicting an increase in QALYs and reductions in costs over a lifetime.

### Disclosures

J.T.C. is an employee of Avalon Health Economics, where J.E.S. is the CEO and principal. Avalon Health Economics was compensated for the completion of this work.

## Supplementary Material

Online Supplementary Material
