# Supplementary material for: Economic Evaluation of a Novel MicroRNA-Based Assay to Determine Risk of Late Genitourinary Radiation Toxicity in Patients With Prostate Cancer
Source: J Health Econ Outcomes Res. 2025 Dec 16;12(2):246–52. doi: 10.36469/001c.146844 (PMC12714312; doi:10.36469/001c.146844)
Supplement: Online Supplementary Material [file jheor_2025_12_2_146844_322576.pdf]

Journal of Health Economics and Outcomes Research

Online Supplementary Material

Economic Evaluation of a Novel MicroRNA-Based Assay to Determine Risk of Late Genitourinary Radiation Toxicity in Patients With Prostate Cancer. *JHEOR*. 2025;12(2):246-252. doi:10.36469/jheor.2025.146844

Table S1: Annual Toxicity Probability by Disease Track

Table S2: Probabilistic Sensitivity Analysis Inputs

Figure S1: Deterministic Sensitivity Analysis Tornado Diagram

Figure S2: Cost-Effectiveness Acceptability Curves

This supplementary material has been provided by the authors to give readers additional information about their work.

This is an open-access article distributed under the terms of the Creative Commons Attribution 4.0 International License (CCBY-4.0). View this license's legal deed at http://creativecommons.org/licenses/by/4.0 and legal code at http://creativecommons.org/licenses/by/4.0/legalcode for more information.

Table S1. Annual Toxicity Probability by Disease Track

Cycle	Rates				
	**Track 1**	**Track 2, 5, 8**	**Track 3,6, 9**	**Track 4**	**Track 7**
1	0.278	0.040	0.119	0.012	0.050
2	0.278	0.040	0.000	0.012	0.050
3	0.278	0.040	0.000	0.012	0.050
4	0.278	0.040	0.000	0.012	0.050
5	0.261	0.037	0.000	0.012	0.047
6	0.245	0.035	0.000	0.011	0.044
7	0.229	0.033	0.000	0.010	0.041
8	0.212	0.030	0.000	0.009	0.038
9	0.196	0.028	0.000	0.009	0.035
10	0.180	0.026	0.000	0.008	0.032
11	0.163	0.023	0.000	0.007	0.029
12	0.147	0.021	0.000	0.007	0.026
13	0.131	0.019	0.000	0.006	0.023
14	0.114	0.016	0.000	0.005	0.021
15	0.098	0.014	0.000	0.004	0.018
16	0.082	0.012	0.000	0.004	0.015
17	0.065	0.009	0.000	0.003	0.012
18	0.049	0.007	0.000	0.002	0.009
19	0.033	0.005	0.000	0.001	0.006
20	0.016	0.002	0.000	0.001	0.003
21	0.000	0.000	0.000	0.000	0.000
22	0.000	0.000	0.000	0.000	0.000
23	0.000	0.000	0.000	0.000	0.000
24	0.000	0.000	0.000	0.000	0.000
25	0.000	0.000	0.000	0.000	0.000
26	0.000	0.000	0.000	0.000	0.000
27	0.000	0.000	0.000	0.000	0.000
28	0.000	0.000	0.000	0.000	0.000
29	0.000	0.000	0.000	0.000	0.000
30	0.000	0.000	0.000	0.000	0.000
31	0.000	0.000	0.000	0.000	0.000
32	0.000	0.000	0.000	0.000	0.000
33	0.000	0.000	0.000	0.000	0.000
34	0.000	0.000	0.000	0.000	0.000
35	0.000	0.000	0.000	0.000	0.000
36	0.000	0.000	0.000	0.000	0.000
37	0.000	0.000	0.000	0.000	0.000
38	0.000	0.000	0.000	0.000	0.000
39	0.000	0.000	0.000	0.000	0.000
40	0.000	0.000	0.000	0.000	0.000
41	0.000	0.000	0.000	0.000	0.000
42	0.000	0.000	0.000	0.000	0.000
43	0.000	0.000	0.000	0.000	0.000
44	0.000	0.000	0.000	0.000	0.000
45	0.000	0.000	0.000	0.000	0.000

Table S1. Annual Toxicity Probability by Disease Track

Cycle	Rates				
	**Track 1**	**Track 2, 5, 8**	**Track 3,6, 9**	**Track 4**	**Track 7**
46	0.000	0.000	0.000	0.000	0.000
47	0.000	0.000	0.000	0.000	0.000
48	0.000	0.000	0.000	0.000	0.000
49	0.000	0.000	0.000	0.000	0.000
50	0.000	0.000	0.000	0.000	0.000
51	0.000	0.000	0.000	0.000	0.000
52	0.000	0.000	0.000	0.000	0.000
53	0.000	0.000	0.000	0.000	0.000
54	0.000	0.000	0.000	0.000	0.000
55	0.000	0.000	0.000	0.000	0.000
56	0.000	0.000	0.000	0.000	0.000
57	0.000	0.000	0.000	0.000	0.000
58	0.000	0.000	0.000	0.000	0.000
59	0.000	0.000	0.000	0.000	0.000
60	0.000	0.000	0.000	0.000	0.000
61	0.000	0.000	0.000	0.000	0.000
62	0.000	0.000	0.000	0.000	0.000
63	0.000	0.000	0.000	0.000	0.000
64	0.000	0.000	0.000	0.000	0.000
65	0.000	0.000	0.000	0.000	0.000
66	0.000	0.000	0.000	0.000	0.000
67	0.000	0.000	0.000	0.000	0.000
68	0.000	0.000	0.000	0.000	0.000
69	0.000	0.000	0.000	0.000	0.000
70	0.000	0.000	0.000	0.000	0.000
71	0.000	0.000	0.000	0.000	0.000
72	0.000	0.000	0.000	0.000	0.000
73	0.000	0.000	0.000	0.000	0.000
74	0.000	0.000	0.000	0.000	0.000
75	0.000	0.000	0.000	0.000	0.000
76	0.000	0.000	0.000	0.000	0.000
77	0.000	0.000	0.000	0.000	0.000
78	0.000	0.000	0.000	0.000	0.000
79	0.000	0.000	0.000	0.000	0.000
80	0.000	0.000	0.000	0.000	0.000
81	0.000	0.000	0.000	0.000	0.000
82	0.000	0.000	0.000	0.000	0.000
83	0.000	0.000	0.000	0.000	0.000
84	0.000	0.000	0.000	0.000	0.000
85	0.000	0.000	0.000	0.000	0.000
86	0.000	0.000	0.000	0.000	0.000
87	0.000	0.000	0.000	0.000	0.000
88	0.000	0.000	0.000	0.000	0.000
89	0.000	0.000	0.000	0.000	0.000
90	0.000	0.000	0.000	0.000	0.000

Table S1. Annual Toxicity Probability by Disease Track

Cycle	Rates				
	**Track 1**	**Track 2, 5, 8**	**Track 3,6, 9**	**Track 4**	**Track 7**
91	0.000	0.000	0.000	0.000	0.000
92	0.000	0.000	0.000	0.000	0.000
93	0.000	0.000	0.000	0.000	0.000
94	0.000	0.000	0.000	0.000	0.000
95	0.000	0.000	0.000	0.000	0.000
96	0.000	0.000	0.000	0.000	0.000
97	0.000	0.000	0.000	0.000	0.000
98	0.000	0.000	0.000	0.000	0.000
99	0.000	0.000	0.000	0.000	0.000
100	0.000	0.000	0.000	0.000	0.000

Table S2. Probabilistic Sensitivity Analysis Inputs

Variable	Mean Value	Standard Error	Distribution
Cost of PROSTOX *ultra*	$4000.00	$400.00	Gamma
Cost of SBRT	$20 670.28	$2067.03	Gamma
Cost of CFRT	$42 642.68	$4264.27	Gamma
Cost of surgery	$97 782.37	$9778.24	Gamma
Cost of care without GU toxicity	$2914.90	$291.49	Gamma
Cost of GU toxicity	$5774.58	$577.46	Gamma
Utility without GU toxicity	0.900	0.130	Beta
Utility of unmanaged GU toxicity	0.698	0.070	Beta
Baseline annual toxicity probability for track 1	0.278	0.028	Beta
Baseline annual toxicity probability for tracks 2, 5, and 8	0.040	0.004	Beta
Baseline annual toxicity probability for track 4	0.012	0.001	Beta
Baseline annual toxicity probability for track 7	0.050	0.005	Beta
Starting age in Markov model, y	67	6.70	Gamma
Annual decrease in toxicity development, %	0.059	0.006	Beta
Percentage of SOC surgical patients using SBRT with low-risk PROSTOX *ultra* result	80.0	0.080	Beta

Abbreviations: CFRT, conventionally fractionated radiation therapy; GU, genitourinary; SBRT, stereotactic body radiation therapy; SOC, standard of care.
Note: Variables not listed in table were assumed to be fixed and were not varied in the probability sensitivity analysis.

Table S1. Deterministic Sensitivity Analysis Tornado Diagram

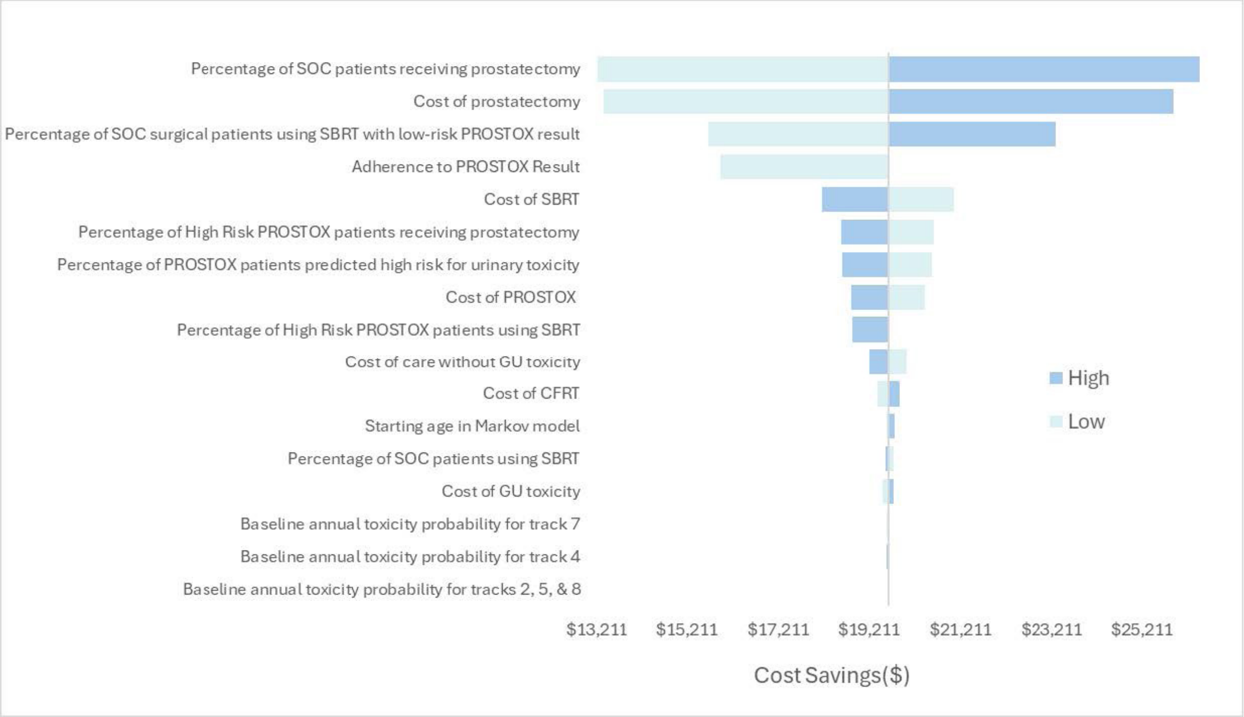

Abbreviations: CFRT, conventionally fractionated radiation therapy; GU, genitourinary; SBRT, stereotactic body radiation therapy; SOC, standard of care.

Table S2. Cost-Effectiveness Acceptability Curves

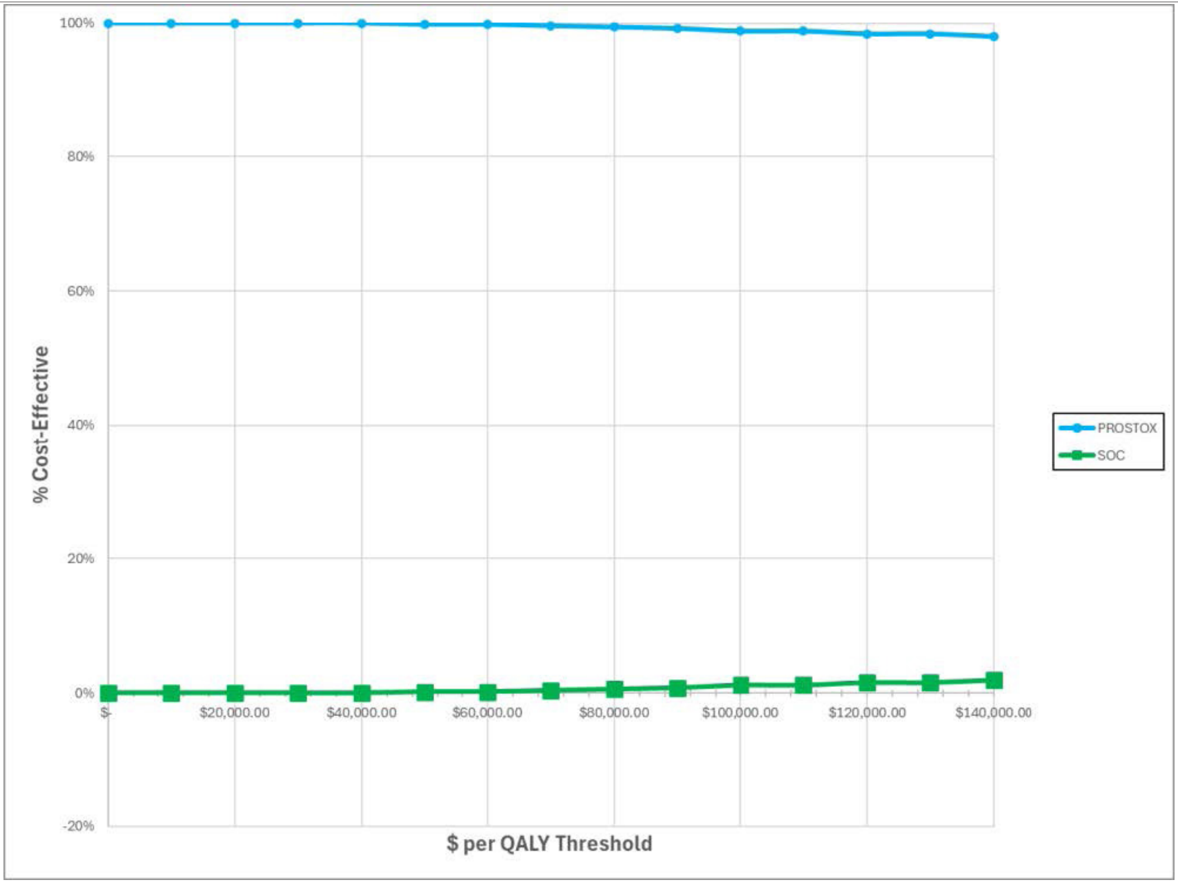

Abbreviations: QALY, quality-adjusted life-year; SOC, standard of care.
